# Macrophages and Fc-receptor interactions contribute to the antitumour activities of the anti-CD40 antibody SGN-40

**DOI:** 10.1038/sj.bjc.6604812

**Published:** 2008-12-09

**Authors:** E Oflazoglu, I J Stone, L Brown, K A Gordon, N van Rooijen, M Jonas, C-L Law, I S Grewal, H-P Gerber

**Affiliations:** 1 Department of Preclinical Therapeutics, Seattle Genetics Inc., Seattle, WA 98021, USA; 2 Department of Antibody Technologies, Seattle Genetics Inc., Seattle, WA 98021, USA; 3 Department of Molecular Cell Biology, Vrije Universiteit, Amsterdam, The Netherlands

**Keywords:** therapeutic antibodies, haematopoietic malignancies, macrophages, non-Hodgkin's lymphoma, antibody-dependent cellular phagocytosis, ADCP, ADCC

## Abstract

SGN-40 is a therapeutic antibody targeting CD40, which induces potent anti-lymphoma activities via direct apoptotic signalling cells and by cell-mediated cytotoxicity. Here we show antibody-dependent cellular phagocytosis (ADCP) by macrophages to contribute significantly to the therapeutic activities and that the antitumour effects of SGN-40 depend on Fc interactions.

CD40 is a member of the tumour necrosis factor (TNF) receptor superfamily and is expressed predominantly on haematopoietic cells, including B-cells, dendritic cells, monocytes, macrophages, activated B-cells and CD8+ T-cells (reviewed in van Kooten and Banchereau (2000)). In lymphoid malignancies, CD40 is present on B-cell precursor acute lymphoblastic leukaemia (ALL (Uckun *et al*, 1990)), non-Hodgkin's lymphoma (NHL (Uckun *et al*, 1990)), Hodgkin's lymphoma (HL (O'Grady *et al*, 1994)) and multiple myeloma (MM (Westendorf *et al*, 1994)).

The humanised monoclonal antibody targeting CD40 (SGN-40) induces potent antitumour effects when tested on CD40-positive tumour cell lines representing a variety of haematologic malignancies (Tai *et al*, 2004; Law *et al*, 2005). The mechanisms mediating the antitumour activity of SGN-40 include direct cytotoxic signalling, inducing caspase-3 activation and apoptosis of tumour cells (Law *et al*, 2005; Tai *et al*, 2005) and natural killer (NK) cell-mediated ADCC. Both, direct cell killing and NK cell-mediated ADCC are enhanced by antibody crosslinking (Law *et al*, 2005) when tested *in vitro*. However, the mechanism involved in mediating anti-lymphoma activity of SGN-40 *in vivo* remained to be investigated.

The ability of several human IgG1-type therapeutic antibodies to engage human effector cells varies significantly, depending on the target antigen and tumour type, and variable degrees of tumour cell death induced by NK cells, neutrophils and macrophages were reported for anti-CD19, -20 and -70 antibodies (reviewed in Tedder *et al* (2006)). Here we show for the first time that macrophages contribute significantly to the antitumour effects of SGN-40 when tested in models of human lymphoma. In addition, a mutant form of SGN-40, lacking Fc-receptor interactions (SGN40-IgG1v1), lacked therapeutic activity when tested *in vitro* and in mice implanted with B-cell lymphomas. The important role of macrophages in mediating therapeutic activity described here may help to guide the clinical development of SGN-40 for the treatment of B-cell malignancies.

## Materials and methods

### Cells and reagents

The CD40-positive lymphoma cell lines Ramos (NHL, Burkitt's lymphoma) and WIL2-S (B-cell lymphoma) were obtained from the American Type Culture Collection (ATCC, Manassas, VA, USA). The CD40-negative cell line L540cy was kindly provided by Dr Phil Thorpe (University of Texas, Southwestern Medical School, Dallas, TX, USA). Cells were grown in RPMI (Life Technologies Inc., Gaithersburg, MD, USA) supplemented with 20% fetal bovine serum (FBS).

### Construction and expression of SGN-40G1v1 variant antibody and Fc*γ* receptors

The anti-CD40 variant containing the mutations, E233P:L234V:L235A (Armour *et al*, 1999) (SGN-40G1v1) was generated using the Quikchange Site-Directed Mutagenesis system (Stratagene, La Jolla, CA, USA). The SGN-40 variant heavy chain and SGN-40 light chain were each cloned into the expression vector pDEF38 (Running Deer and Allison, 2004) downstream of the CHEF EF-1*α* promoter and stably expressed in CHO-DG44 (Urlaub *et al*, 1986) cells. SGN-40 and SGN-40G1v1 proteins were expressed in CHO-DG44 cell lines and purified by protein A chromatography. Complementary DNA clones for human (hu) Fc*γ*RI (IMAGE clone 5248549), huFc*γ*RIIIA V158 (IMAGE clone 5206097) and huFc*ε*R *γ* chain (IMAGE clone 5219148) were obtained from Invitrogen (Carlsbad, CA, USA) and coding regions were introduced into a mammalian expression vector system. Proteins were expressed in CHO-DG44 cell lines and highly expressing clones were selected by FACS and recovered by limited dilution cloning. Preliminary pharmacokinetic analysis of serum samples from treated mice revealed comparable characteristics between SGN-40 and SGN-40G1v1 (data not shown).

### *In vitro* characterisation of antibody binding

SGN-40 was labelled with Alexa Fluor 488 carboxylic acid–succinimidyl ester conjugation using the Invitrogen Alexa Fluor 488 labeling kit (Invitrogen). For binding experiments, Ramos or stable CHO DG-44 cells expressing huFc*γ*RI and huFc*γ*RIIIA V158 were mixed and combined with serial dilutions of SGN-40 or the SGN-40G1v1 variant. Labelled cells were detected using an LSRII FACS analyzer (Beckton Dickinson Biosciences, San Jose, CA, USA). The flow cytometry data was analysed with a one site competition model equation using Prism v4.01 (GraphPad Software, San Diego, CA, USA).

### Antibody-dependent cellular phagocytosis assay

The assay was performed as described earlier (McEarchern *et al*, 2007). Briefly, after labelling of CD40-positive target cells (Ramos), cells were pre-coated with SGN-40 or SGN-40G1v1 and incubated with monocyte-derived macrophages. The purity of the macrophage preparations were routinely >95% (data not shown).

### Xenograft models and effector cell ablation and IHC analysis

Tumour bearing CB-17/lcr SCID mice were depleted of effector cells by using anti-asialo-GM 1 (1.25 mg kg^−1^, neutrophils) and anti-Gr-1 (4 mg kg^−1^, NK cells) clodronate encapsuled liposomes (CEL, macrophage), as described earlier (Oflazoglu *et al*, 2007). For IHC analysis, liver and spleen sections were stained with an anti-mouse F4/80 (macrophage marker) (AbD Serotec, Oxford, UK) using the Bondmax autostainer (Leica Microsystems Inc., Bannockburn, IL, USA) with an alkaline phosphatase-fast red detection kit. Mice were monitored at least twice per week and were sacrificed when they exhibited signs of disease, including weight loss of 15–20%, hunched posture, lethargy, cranial swelling or dehydration. Statistical analysis was conducted using the log-rank test with the Graphpad Prism Software Package version 4.01 (Graphpad). All animal experiments were performed according to the guidelines of the Institutional Animal Care and Use Committee (IACUC).

## Results and discussion

**Figure 1:**
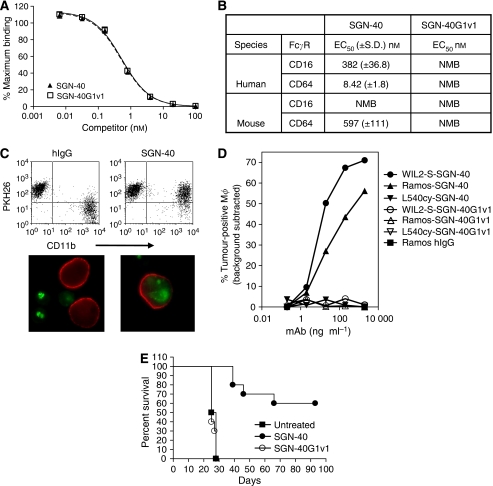
SGN-40 mediates antibody-dependent cellular phagocytosis (ADCP) activity and requires functional Fc–Fc*γ*R interaction *in vitro*. (**A**) Binding of SGN-40 and SGN-40G1v1 to CD40+ Ramos cells was detected by flow cytometry. EC_50_ values are 0.510 and 0.528 nM for SGN-40 and SGN-40G1v1, respectively. (**B**) Binding of SGN-40 and SGN-40G1v1 to CHO DG-44 cell lines expressing human or mouse Fc*γ*RI (CD64) and Fc*γ*RIIIA V158 (CD16) as determined by flow cytometry. Data is reported as the percent of maximum fluorescence, calculated by the sample fluorescence divided by the fluorescence of cells stained with anti-CD40-Alexa Fluor 488. NMB=non-measurable binding. (**C**) SGN-40 induces ADCP activity *in vitro* as determined by flow cytometry and fluorescence microscopy. Ramos target cells were labelled with PKH26 lipophilic dye for tracking purposes, and treated with non-binding control IgG or SGN-40 MAb and mixed with human monocyte-derived macrophages (Mø). Mø were stained with PE-conjugated anti-CD11b. Cells present in the upper right quadrant (PKH26+CD11b+) are Mø that internalised tumour cells. For fluorescence microscopy, tumour cells were labelled with PKH67 (green) and the macrophages were detected with Alexa Fluor 568-conjugated antibody specific for CD11b (red). No ADCP activity was detected on control, CD40-negative Hodgkin's lymphoma (HL) cells L540cy (**D**) CD40-positive Ramos, WIL2-S and the CD40-negative L540cy target cells were labelled with PKH26 lipophilic dye, treated with varying concentrations of SGN-40, SGN-40G1v1 or non-binding control IgG then mixed with Mø. (**E**) Survival curve of mice implanted with Ramos tumour cells and left untreated or following treatment with 4 mg kg^−1^ SGN-40 or SGN-40G1v1 on day 1 (*n*=10 per group), untreated *vs* SGN-40 (*P*<0.0001), untreated *vs* SGN-40G1v1 (*P*=0.7190), SGN-40 *vs* SGN-40G1v1 (*P*<0.0001). Data shown are from one representative of a total of two independent experiments.

To study the contributions of Fc-receptor interactions to the antitumour activity of SGN-40, we constructed an Fc variant form of SGN-40 containing a substitution of three amino acids in the Fc portion of the *γ*1 heavy chain (SGN-40G1v1). When introduced to other therapeutic MAbs of the IgG1 isotype, these changes resulted in abolished Fc-receptor interactions (Armour *et al*, 1999). As expected, no changes in the ability of SGN-40 and SGN-40G1v1 to bind to CD40 were observed (Figure 1A). However, competition binding assays with cells expressing human or mouse CD16 (Fc*γ*RIII) and CD64 (Fc*γ*RI) confirmed that SGN-40G1v1 lacked binding affinity to Fc receptors (Figure 1B). For SGN-40, weaker binding to mouse relative to human CD64 (Fc*γ*R1) and lack of binding to mouse CD16 (Fc*γ*RIII) were also noticed. Although Fc*γ*-receptor interactions are essential for effector cell activation, no direct correlation between binding affinities of therapeutic antibodies and activation of effector cells were reported (Richards *et al*, 2008), because these responses are ultimately regulated by the balance between activating and inhibitory signals delivered through Fc*γ* receptors. Therefore, the differences in binding affinity of SGN-40 between Fc*γ*-RI+III are not predictive for antitumour activities *in vivo*.

To determine the levels of antibody-dependent cellular phagocytosis (ADCP) engagement, SGN-40 and SGN-40G1v1 were incubated in presence of primary human monocytic cells and tested for their ability to induce phagocytosis of CD40+ Ramos (Burkitt's lymphoma, Figures 1C and D) and WIL2-S cells (B-cell lymphoma, Figure 1D) by macrophages. Although SGN-40 induced robust ADCP, SGN-40G1v1 was devoid of detectable ADCP activity, showing that Fc–Fc*γ*-receptor interactions are necessary for phagocytic uptake of target lymphoma cells (Figure 1D). These studies show that Fc-dependent ADCP may represent an important mechanism by which SGN-40 induces anti-lymphoma effects.

**Figure 2:**
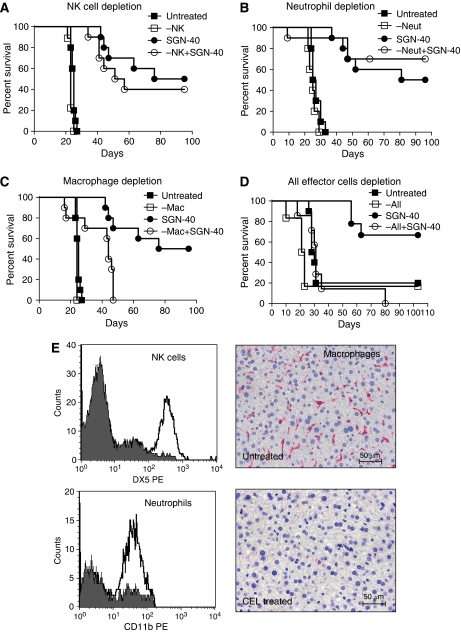
Macrophages mediate antitumour activity of SGN-40 and requirement for intact Fc–Fc*γ*R interactions *in vivo*. (**A**) Mice implanted with Ramos tumours (NHL) were depleted of natural killer cells (−NK) (**B**) neutrophils (−Neut), (**C**) macrophages (−Mac) or (**D**) all subsets combined (−All). Mice were either left untreated or treated with 4 mg kg^−1^ SGN-40 on day 1 post tumour implantation (*n*=10 per group), using intraperitoneal injections. Untreated *vs* SGN-40 (*P*<0.0001), untreated *vs* −NK+SGN-40 (*P*<0.0001), SGN-40 *vs* −NK+SGN-40 (*P*=0.4611). Untreated *vs* −Neut+SGN-40 (*P*=0.0001), SGN-40 *vs* −Neut+SGN-40 (*P*=0.5969). Untreated *vs* −Mac+SGN-40 (*P*=0.002), SGN-40 *vs* −Mac+SGN-40 (*P*=0.0016). Untreated *vs* All depleted (*P*=0.0594), SGN-40 *vs* All depleted +SGN-40 (*P*=0.0004). Data shown are from one representative of a total of two or three independent experiments. (**E**) FACS analysis of spleen from NK cell-depleted mice using anti-DX5 antibody, or peripheral blood from neutrophil-depleted mice, using an anti-CD11b antibody. The right panels display IHC analysis of liver tissues isolated from macrophage-depleted mice using an anti-mouse-F4/80 antibody.

To investigate the relevance of Fc–Fc*γ*R interactions for overall therapeutic activity, we compared survival of tumour bearing mice treated with either SGN-40 or SGN-40G1v1. In contrast to SGN-40, SGN-40G1v1 did not delay disease progression relative to untreated mice (Figure 1E). These findings indicate that all mechanisms contributing to anti-lymphoma activities by SGN-40 are dependent on Fc interactions in this NHL model, including direct apoptotic signalling in tumour cells. Next, we were interested to identify the identity of the effector cells mediating the antitumour activity of SGN-40 *in vivo*. For this purpose, tumour-bearing mice were selectively depleted of either NK cells (Figure 2A), neutrophils (Figure 2B), macrophages (Figure 2C) or all three cell types combined (Figure 2D). Although the lack of NK cells and neutrophils did not significantly affect the antitumour activity of SGN-40, depletion of macrophages, either alone or combined with NK and neutrophil cells, resulted in decreased survival of tumour-bearing mice compared with non-effector cell ablated animals (Figures 2C and D, *P*=0.0016 and 0.0004, respectively). These findings are in agreement with an earlier report showing potent antitumour activity of SGN-40 in SCID-beige mice, which display impaired NK cell functions (Law *et al*, 2005). Antitumour activity was evident in macrophage ablated mice (Figure 2C), suggesting that non-macrophage-dependent mechanisms may contribute to the *in vivo* activity in this model, including direct apoptotic signalling in tumour cells. The degree of effector cell depletion was monitored by FACS or IHC analysis (Figure 2E). Robust target effector cell depletion was confirmed by flow cytometric analysis of splenocytes (NK cells, anti-DX5), peripheral blood (neutrophils, anti-CD11b) or by F4/80-IHC staining (macrophages) in liver sections.

In humans, macrophages express all three Fc*γ* receptors, whereas neutrophils and NK cells express predominantly Fc*γ*RII and Fc*γ*RIII (Koene *et al*, 1997). Sequence nucleotide polymorphisms within the *FCGR2* and *FCGR3* loci in humans alter their binding affinities to IgG1 Mabs, and ultimately impact on downstream effector cell engagement of therapeutic Mabs such as the anti-CD20 antibody Rituximab (Weng and Levy, 2003). In addition, the contributions of effector cells to antitumour effects of therapeutic MAbs were shown to vary between tumour types. For example, Fc*γ*R II+III single nucleotide polymorphisms (SNPs) correlated with therapeutic activity of rituximab in NHL, but not in CLL patients (Cartron *et al*, 2002; Weng and Levy, 2003; Farag *et al*, 2004). Combined, these findings suggest that the analysis of patient Fc*γ*-receptor polymorphisms and comparison with clinical response rates may provide important information regarding the mechanism of action employed by SGN-40 in different heme-malignancies, including NHL and MM. Importantly, the chemotherapy agents used for the treatment of NHL were shown to interfere differentially with macrophage functions (Marcus and Hagenbeek, 2007). Given the important role of macrophages in mediating the therapeutic activity of SGN-40 described in this report, it is tempting to speculate that combining SGN-40 with therapeutic agents that enhance macrophage activities, including Revlimid in MM indications, may be beneficial.
